# Application of the New IMWG/IMS High-Risk Classification for Multiple Myeloma: Analysis of a Large Real-World Romanian Cohort

**DOI:** 10.3390/ijms27104620

**Published:** 2026-05-21

**Authors:** Sorina Nicoleta Badelita, Sinziana Barbu, Onda-Tabita Calugaru, Cerasela Jardan, Codruta Delia Popa, Larisa Zidaru, Mihai Emanuel Himcinschi, Bogdan Nicolas Smadu, Iulia Ursuleac, Daniel Coriu

**Affiliations:** 1 Clinical Department I, Hematology, Fundeni Clinical Institute, 022328 Bucharest, Romania; sorinabadelita@gmail.com (S.N.B.); delia.popa@umfcd.ro (C.D.P.); emilia-larisa.zidaru@drd.umfcd.ro (L.Z.); iulia.ursuleac@umfcd.ro (I.U.); daniel.coriu@umfcd.ro (D.C.); 2 Hematology Department, “Carol Davila” University of Medicine and Pharmacy, 020022 Bucharest, Romania; mhimcinschi@gmail.com (M.E.H.);; 3 Biochemistry Department, “Victor Babes” University of Medicine and Pharmacy, 300041 Timisoara, Romania

**Keywords:** multiple myeloma, high-risk disease, cytogenetic risk stratification, fluorescence in situ hybridization, del(17p), chromosome 1 abnormalities, tandem autologous stem cell transplantation

## Abstract

Multiple myeloma (MM) is a biologically heterogeneous plasma cell malignancy in which prognosis is strongly influenced by cytogenetic abnormalities. Recent updates from the International Myeloma Working Group (IMWG), along with the European Hematology Association (EHA) and European Myeloma Network (EMN), have refined the definition of high-risk (HR) disease by integrating TP53 alterations, chromosome 1 abnormalities, and specific combinations of cytogenetic lesions. However, validation of these criteria in real-world patient populations remains limited. We conducted a retrospective, single-center study including 738 patients diagnosed with MM between 2017 and 2025, of whom 408 had available fluorescence in situ hybridization (FISH) data at diagnosis. Patients were reclassified according to the latest IMWG/IMS high-risk criteria proposed in international literature. Cytogenetic abnormalities, treatment patterns, and clinical outcomes, including overall survival (OS), progression-free survival (PFS), response rates, and relapse, were analyzed. Survival was estimated using the Kaplan–Meier method. A total of 103 patients (25%) were reclassified as high-risk according to IMWG/IMS high-risk criteria. Cytogenetic HR abnormalities were identified in 17.2% of cases, with del(17p) being the most frequent (14.7%). Median OS and PFS in HR patients were 52.4 months and 16 months, respectively, compared with 68.4 months and 28 months in standard-risk patients (log-rank test *p* values of 0.0197 and 0.0004, respectively). Although overall response rates were high (83% in HR vs. 91% in standard-risk), relapse remained frequent in HR patients. Outcomes varied significantly according to cytogenetic complexity. Isolated del(17p) was associated with improved survival compared with cases harboring additional abnormalities, while double-hit and triple-hit profiles demonstrated inferior outcomes. The presence of chromosome 1 abnormalities, particularly in combination with IGH translocations, further worsened prognosis. Among HR patients, 44% underwent autologous stem cell transplantation (ASCT), including 10 cases of TANDEM ASCT. No survival benefit was observed for TANDEM compared with single ASCT, with median OS of 52.9 vs. 78.3 months, respectively (log-rank test *p* values of 0.2516). Our real-world analysis supports the prognostic relevance of the updated IMS/IMWG high-risk criteria in MM. Cytogenetic complexity, rather than individual abnormalities alone, is a key determinant of outcome. Despite high response rates achieved with modern therapies, survival remains inferior in HR patients. TANDEM ASCT did not confer additional benefit in this cohort, supporting a more individualized approach to treatment intensification.

## 1. Introduction

Multiple myeloma is a hematologic neoplasia characterized by monoclonal proliferation of plasma cells [1]. This heterogeneity requires rigorous risk stratification to assess patient prognosis and establish further therapeutic strategies [2]. The initial risk assessment algorithm for patients with multiple myeloma was based on the ISS stage, which included β2-microglobulin and albumin (B2M) [3]. This staging did not consider the genetic basis of the disease, underestimating the risk class of a substantial number of patients. In 2015, the staging system was updated (Revised International Staging System, R-ISS) to include LDH level values and cytogenetic anomalies associated with unfavorable evolution, namely del(17p); t(4;14) and t(14;16) [4]. The R-ISS system, currently used on a large scale for risk staging, integrates ISS standardization with FISH cytogenetics and LDH level values, yielding a three-tier risk classification. Even with this addition to the diagnostic criteria of MM, a certain subset of patients continued to have an unfavorable evolution despite not meeting the criteria [5]. The last few years have demonstrated that, on the one hand, other chromosomal anomalies, especially in the context of coexistence, can negatively affect the prognosis; on the other hand, some previously considered high-risk conditions now require association with other modifications to be regarded as such [6].

Therefore, in 2022, EMN proposed a revision of the R-ISS staging system. R2-ISS stages patients into 4 risk categories and includes the 1q gain criterion as a high-risk factor [7]. In the initial R-ISS version, stage II comprised a highly heterogeneous patient population. The updated model reclassified a subset of these patients into higher-risk stages by integrating the 1q gain criterion.

In 2025, updated recommendations regarding the definition of high-risk multiple myeloma (MM) were proposed by the International Myeloma Society/International Myeloma Working Group (IMS/IMWG) and further discussed within the EHA-EMN guidelines for the diagnosis, treatment, and follow-up of MM patients. According to the current IMWG/IMS high-risk criteria, high-risk MM is defined by the presence of at least one of the following: del(17p) or a TP53 alteration; an IGH translocation [t(4;14), t(14;16), or t(14;20)] associated with chromosome 1 abnormalities (1q gain/amplification or 1p deletion); significant chromosome 1 alterations (biallelic del(1p) or the combination of monoallelic del(1p) and gain(1q)); or a serum β2-microglobulin level ≥ 5.5 mg/L in the absence of renal impairment [6,8].

The main changes introduced were: the addition of TP53 mutations, recognizing their major prognostic impact; reevaluation of IGH translocations as high-risk factors only when concurrent with another cytogenetic modification; the rising importance of chromosome 1 cytogenetic anomalies, whether deletion, gain, or amplification; and the recognition of β2-microglobulin as an independent prognostic factor, highlighting that a large tumor burden leads to an unfavorable prognosis even in the absence of cytogenetic modifications.

The improvements brought to staging systems for patients with multiple myeloma reshaped patient management as early as the diagnosis stage. Initial evaluation should include a complete, standardized workup incorporating FISH testing to assess high-risk cytogenetic abnormalities, along with TP53 mutation analysis. The identification of HR MM patients is essential, having an impact on both therapeutic conduct and further disease monitoring (MRD). For first-line treatment, the new EHA-EMN guidelines maintain a distinct, more intensive therapeutic approach for HR patients compared to standard risk, even in the era of modern therapies [9].

Despite substantial progress made in recent years, a number of limitations persist in the current MM staging literature, underscoring the need for further analysis. A major shortcoming of the current literature is the lack of validation in real-world patient cohorts. The new HR criteria in MM (2025) are derived primarily from retrospective data of clinical trials on pre-selected patient populations; their prognostic value has not been fully validated in heterogeneous, real-world settings with variable therapeutic approaches [10,11].

Although the introduction of monoclonal antibodies and quadruplet regimens has led to significant progress in the clinical course of patients with multiple myeloma [12], data regarding the efficacy of these therapeutic strategies in ultra-high-risk patients in real-world settings remains limited [13]. In many clinical trials, these patient subgroups are underrepresented, thereby limiting the robustness of statistical analyses. Consequently, in the absence of data from large cohorts of patients treated in real-world settings, it becomes difficult to assess the true benefit of these therapies for very high-risk patients [14].

In this context, the objective of our study was to retrospectively reclassify a large cohort of patients with multiple myeloma according to the new IMWG/IMS HR criteria and to analyze the impact of this new staging system on OS and PFS.

## 2. Results

Between 2017 and 2025, 738 patients with multiple myeloma were diagnosed at the Department of Hematology of the Fundeni Clinical Institute. Cytogenetic analysis using FISH was performed in 408 patients (55.3%) at the time of diagnosis of multiple myeloma, and these patients constituted the study cohort. Of these, 103 patients (25%) were reclassified into the HR category, according to the IMWG/IMS HR criteria (5,6), either due to the presence of cytogenetic abnormalities with an unfavorable prognosis identified by FISH analysis or due to elevated B2M levels (≥5.5 mg/L and creatinine level < 1.2 mg/dL). FISH analysis identified 70 cases with high-risk cytogenetic abnormalities, according to the IMWG/IMS HR criteria [5,6]. In addition, 33 patients were classified as HR based solely on elevated B2M levels (according to IMWG/IMS [5,6]). The cytogenetic abnormalities detected by FISH analysis are summarized in Table 1.

In the subgroup comprising HR patients (n = 103), a higher proportion was observed among younger patients (<65 years); these accounted for 72% of all patients (n = 74), with a slight male predominance (51%). Most patients (66%; n = 68) had stage III disease according to the ISS classification at diagnosis. The general characteristics of the patients are summarized in Table 2.

**Figure 1:**
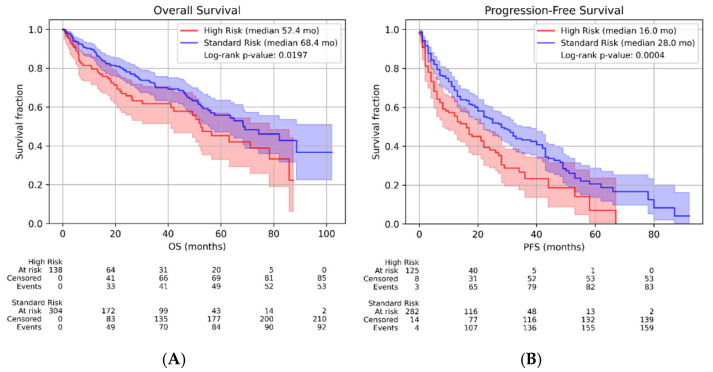
(**A**) and (**B**): Overall survival (OS) (**A**) and progression-free survival (**B**) in HR (high-risk patients) and SR (standard-risk patients). Kaplan–Meier curves illustrating OS (**A**) and first-line treatment PFS (**B**) according to risk group. Median follow-up HR: 29.1 months. Median follow-up SR: 37.6 months.

The subsequent analysis focused on evaluating survival parameters (PFS and OS) and treatment response by comparing the two subgroups within the study cohort: high-risk (HR) patients and standard-risk (SR) patients. Therefore, in the HR subgroup, the median OS was 52.4 months, and the median first-line progression-free survival (PFS) from treatment initiation was 16 months. For this group, the ORR to first-line treatment was 83%, while the relapse rate was 31%. In the SR subgroup, the median OS was 68.4 months, and the median first-line PFS was 28 months. The ORR to first-line treatment in this group was 91%, with a relapse rate of 27%. The Kaplan–Meier curves stratified by risk category for OS and first-line PFS are shown in Figure 1A and Figure 1B, respectively.

Regarding the treatment strategy, most patients received combination therapies that included proteasome inhibitors (PI), immunomodulatory drugs (IMiD), and anti-CD38 antibodies. Thus, 85% (n = 88) of patients were treated with bortezomib, 86% (n = 89) with lenalidomide, and 37% (n = 38) with daratumumab. These therapeutic combinations resulted in an overall response rate (ORR) of 83%, with 55% achieving at least VGPR. When eligible, autologous stem cell transplantation was also performed. Among HR patients, 44% (n = 45) underwent at least one autologous transplant: 35 received a single ASCT after first-line treatment, while 10 underwent ASCT in TANDEM. For most patients, the TANDEM procedure was not performed due to insufficient stem cell harvest. Among HR patients who underwent upfront single ASCT (n = 35), median OS and PFS were 78 and 44 months, respectively. In contrast, patients treated with tandem ASCT (n = 10) had a median OS of 52 months and a median PFS of 21 months, while maintaining high rates of VGPR and CR following induction therapy. After induction and ASCT, many of these patients (77%) received maintenance therapy with bortezomib given alone or in association with lenalidomide.

It is important to note that before reclassifying patients as HR per the latest IMWG/IMS HR criteria, and within the same cohort (n = 408) with available FISH analysis over the 8-year period (2017–2025), the initial high cytogenetic risk group included 145 patients. The old inclusion criteria for cytogenetic HR were: del(17p) ≥ 10%, t(4;14) or t(14;16), or 1q+ in combination with one of these abnormalities.

Of these 145 patients, 47 underwent a single ASCT, and 22 received TANDEM ASCT. Notably, in this cohort classified as HR under the old criteria, TANDEM transplantation did not improve overall survival. The median OS was 52.8 months for patients who underwent TANDEM ASCT, compared with a median OS not yet reached for those who received only a single ASCT.

**Figure 2:**
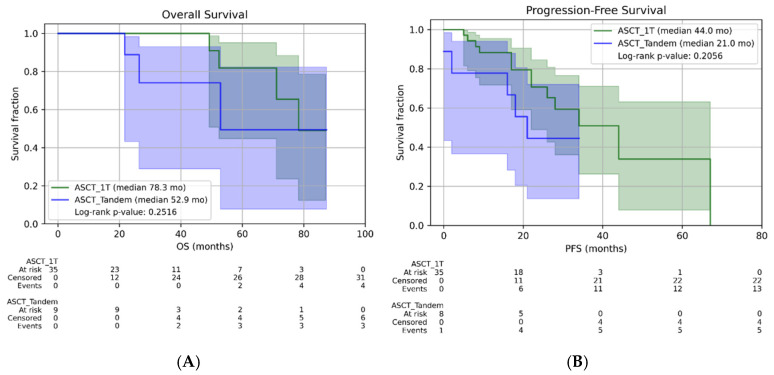
(**A**) and (**B**): Overall survival (OS) (**A**) and progression-free survival (**B**) in HR (high-risk patients). Kaplan–Meier illustrating OS (**A**) and PFS (**B**) according to transplant strategy (single vs. TANDEM). Median follow-up ASCT 1T: 29.1 months and median follow-up ASCT Tandem: 34.6 months.

Even in the cohort of patients reclassified as HR (n = 103), the addition of a second ASCT (TANDEM) did not confer any benefit in OS or PFS, as shown by the Kaplan–Meier curves (Figure 2A,B). Patients who underwent a single autologous stem cell transplant (n = 35) achieved a median OS of 78.3 months and a median PFS of 44 months, compared with a median OS of 52.9 months and a median PFS of 21 months in the TANDEM group. (n = 10). Regarding the overall response rate and the depth of responses achieved after first-line treatment (induction, transplantation, and maintenance), the results are presented comparatively in Table 3. The data suggest that the overall response rate is higher in patients who underwent a single transplant procedure (97% vs. 90%). Additionally, for this same patient group, there is a trend toward deeper responses (37% of patients with a single ASCT procedure achieved CR).

In the cohort of patients who underwent FISH (n = 408), several HR cytogenetic abnormalities were identified. The 17p deletion was identified in 60 patients (15%). In this subgroup, median OS was 52.9 months, median PFS was 18 months, ORR for first-line treatment was 85%, 12-month survival rate was 82%, and males predominated (60%). Of the 60 patients with del 17p, 9 underwent ASCT in TANDEM.

Within the cohort of patients fulfilling the current IMWG/IMS high-risk definition, additional cytogenetic lesions, involving chromosome 1 abnormalities or concurrent TP53-associated alterations, appeared to further worsen clinical outcomes, supporting the concept that cytogenetic complexity contributes to disease aggressiveness beyond isolated lesions.

The association between cytogenetic alterations involving chromosomes 14 and 1 was detected in 12 patients, of whom 10 (2.5%) had t(4;14) and 2 (1%) had t(14;16). In both subgroups, a predominance of female patients was observed. In the case of the association of chromosome 1 abnormalities with t(4;14), median OS had not been reached at the time of analysis, and median PFS was 13 months, suggesting limited disease control. The ORR in the first-line treatment was 67%, and the disease recurrence rate was 40%. In the cohort of 408 patients, the association of del(1p) and 1q+ was identified in 2 patients (1%). Although the response rate to first-line treatment was 100%, subsequent progression was unfavorable.

Similarly, del(1p) was identified in 5 patients (1%). This subgroup was predominantly male (60%), with a treatment response rate of 100%, median OS and PFS not reached, and a first-line treatment response rate of 100%. Although outcomes appeared numerically favorable in this very small subgroup, the limited number of patients precludes definitive prognostic interpretation.

In the analyzed cohort, del(17p) was the most common HR cytogenetic abnormality detected by FISH (15%); the remaining HR abnormalities (per IMWG/IMS) were encountered in isolation. The response to first-line treatment (ORR), although achieved at high rates across most patient subgroups, did not translate into long-term disease control. The comparative results of these cytogenetic alterations are detailed in Table 4.

Within the analyzed cohort, patients classified as HR were subsequently stratified based on the number of adverse cytogenetic abnormalities detected. Patients with a single cytogenetic abnormality were defined as single-hit, those with two concurrent abnormalities as double-hit, and those with three or more high-risk cytogenetic abnormalities were classified as triple-hit.

Analysis of these data revealed a predominance of the double-hit category, with 64 patients. The most common associations in this category were del(17p) and 1q+ (22 patients) and 1q+ and t(4;14) (10 patients). A total of 9 patients were classified as triple-hit, presenting an association of at least 3 FISH abnormalities, among which del(17p) was very common. There was no association between del(1p) and alterations in chromosome 14.

**Figure 3:**
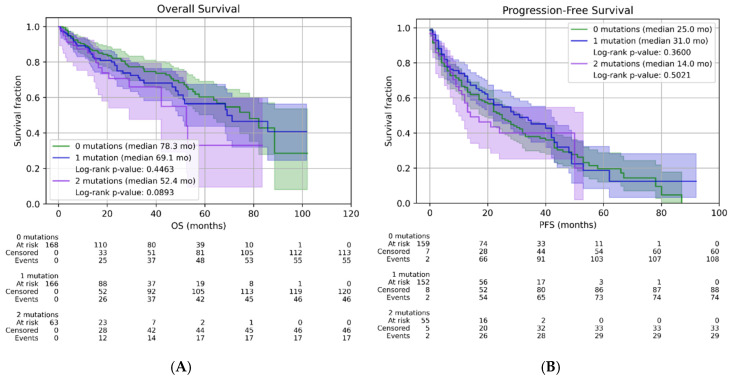
(**A**) and (**B**): Kaplan–Meier curves for overall survival (OS) (**A**) and progression-free survival (PFS) (**B**) according to the number of FISH abnormalities.

Analysis of the data revealed that the longest progression-free survival (PFS) was achieved in the subgroup with del(17p) associated with 1q+, with a median PFS of 24 months; whereas the longest survival was observed in the group with 1q+ and t(4;14), where median OS was not reached. Another important observation is that the presence of t(14;16), particularly in triple combinations, was associated with an unfavorable prognosis. The information on HR cytogenetic subgroups is summarized in Table 4, and the survival data are presented for comparison in Figure 3A,B.

Median follow-up 0 mutations: 50.6 months; Median follow-up 1 HR cytogenetic abnormality: 26.8 months; Median follow-up 2 HR cytogenetic abnormalities: 18.6 months.

**Figure 4:**
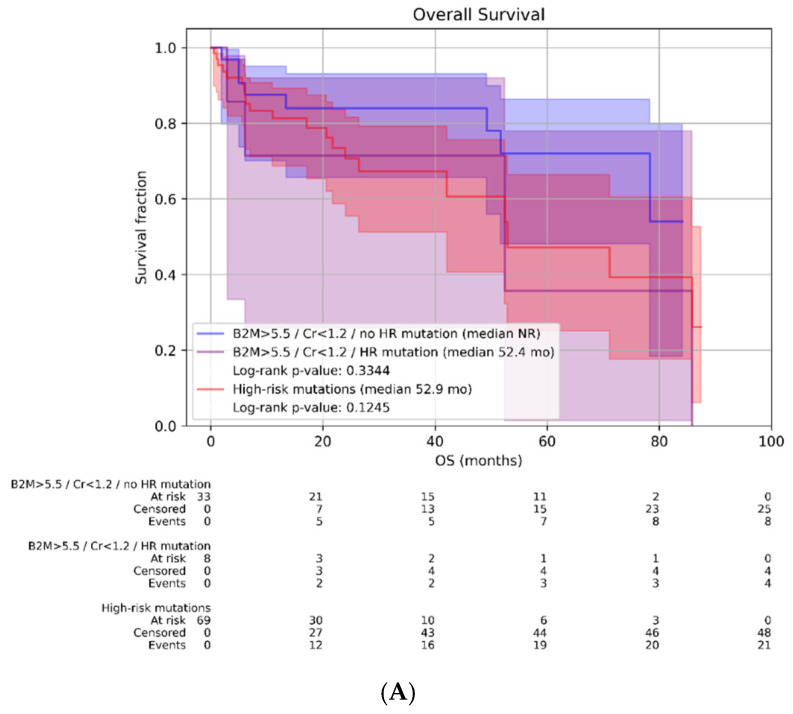

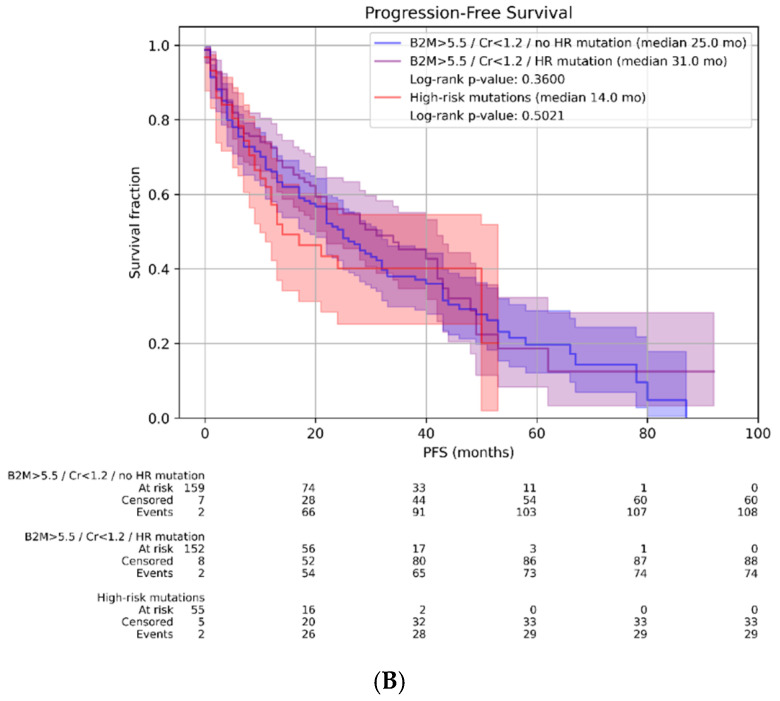
(**A**) Kaplan–Meier curves for OS according to HR criteria (cytogenetic vs. B2M). (**B**) Kaplan–Meier curves for PFS according to HR criteria (cytogenetic vs. B2M).

To assess the impact of the comprehensive definition of high risk according to the latest IMWG/IMS guidelines, we compared survival in patients classified as high risk based on elevated B2M levels without renal impairment. As shown in Figure 4A,B, the Kaplan–Meier analysis highlights better survival outcomes in patients classified as HR based on high tumor burden (B2M ≥ 5.5 mg/L and creatinine < 1.2 mg/dL), but without high-risk cytogenetic alterations according to IMWG/IMS. The combination of these factors negatively impacts PFS and OS. Overall, stratification based on these factors did not demonstrate a significant impact on survival.

Median follow-up B2M ≥ 5.5 mg/L/ Cr < 1.2 mg/dL/no HR cytogenetic abnormalities: 57.2 months; Median follow-up B2M ≥ 5.5 mg/L/Cr < 1.2 mg/dL/HR cytogenetic abnormality: 36.3 months; Median follow-up HR cytogenetic abnormalities: 25.6 months.

## 3. Discussion

Multiple myeloma is a biologically heterogeneous plasma cell malignancy, with prognosis largely driven by cytogenetic abnormalities and disease stage. Risk stratification based on fluorescence in situ hybridization (FISH), as recommended by the International Myeloma Working Group, identifies high-risk lesions such as del(17p), t(4;14), t(14;16), and chromosome 1 abnormalities, which are consistently associated with inferior outcomes. Despite advances in therapy, including proteasome inhibitors, immunomodulatory drugs, monoclonal antibodies, and autologous stem cell transplantation (ASCT), patients with high-risk cytogenetic features continue to have poorer survival [15].In this context, we analyzed a cohort of 408 patients with multiple myeloma from a tertiary hematology center, focusing on the prevalence of high-risk cytogenetic abnormalities and their impact on clinical outcomes, including overall survival, progression-free survival, response rates, and relapse patterns.

In the present cohort, 17.2% of patients were classified as high-risk based on cytogenetic abnormalities, a proportion slightly lower than the 20–30% typically reported in large international datasets. The distribution of high-risk cytogenetic abnormalities in our cohort differed slightly from that reported in larger international datasets, such as the NDMM cohort described by Schavgoulidze et al., in which combinations involving IGH translocations and chromosome 1 abnormalities were more frequent [16]. Several factors may explain this discrepancy. First, our study is a retrospective, single-center real-world cohort characterized by heterogeneous treatment periods and variable access to comprehensive cytogenetic testing. Second, complete characterization of chromosome 1 abnormalities was not uniformly available in all patients, particularly regarding gain versus amplification of 1q and monoallelic versus biallelic del(1p). In addition, TP53 mutational testing was not systematically performed. Finally, only a subset of the initially diagnosed population had a complete baseline FISH evaluation available, which may also have influenced the observed cytogenetic distribution. These findings highlight the practical challenges of implementing comprehensive, modern risk stratification in routine clinical practice. This difference may reflect cohort composition and classification criteria, as well as the distribution of individual cytogenetic abnormalities.

Among high-risk patients, del(17p) was the most frequent abnormality, identified in 14.7% of all cases, which is consistent with the prevalence reported in the literature (approximately 8–15%).

Patients harboring del(17p) demonstrated a median OS of 52.9 months and PFS of 18 months, closely aligning with published real-world data (OS~47–52 months, PFS~21–30 months) [17]. The overall response rate (85.2%) was comparable to literature values, reflecting the effectiveness of modern therapeutic regimens; however, relapse remained frequent (46.7%), confirming the adverse prognostic impact of TP53 disruption.

Interestingly, patients with isolated del(17p) in our cohort showed a longer median OS (85.8 months) compared with the overall del(17p) group, suggesting that co-occurring cytogenetic abnormalities significantly modulate prognosis, a finding also supported by previous studies [15,18].

The presence of additional cytogenetic abnormalities, particularly 1q gain, was associated with inferior outcomes. Patients with del(17p) + 1q+ (5.4% of the cohort) had a median OS of 42.1 months and PFS of 24 months, compared with literature data indicating OS values ranging from approximately 29 to 36 months and PFS between 15 and 25 months for double-hit disease [15]. Although response rates remained relatively high (82.4%), the presence of double high-risk anomalies reflects a biologically more aggressive disease, consistent with previously described double-hit myeloma.

Other high-risk combinations further illustrated this pattern. Patients with del(17p) + t(4;14) exhibited markedly poor outcomes, with a median OS of 20.6 months and PFS of 10 months, in line with published data [19]. Similarly, combinations involving t(4;14) + 1q+ were associated with short PFS (13 months) and reduced response rates (66.7%), highlighting the aggressive nature of these cytogenetic profiles [20].

Although rare, del(17p) + t(14;16) cases showed extremely high relapse rates (100%), supporting existing evidence that this subgroup carries a particularly unfavorable prognosis [15,17].

Triple-hit abnormalities were rare in our cohort (<1%) but consistently associated with extremely poor outcomes, including rapid relapse and very short survival. Although median OS and PFS could not be reliably estimated due to small numbers, these findings are consistent with literature data showing that the accumulation of ≥3 high-risk cytogenetic events leads to dramatically reduced survival [15].

The cumulative effect of cytogenetic abnormalities was further demonstrated by stratifying patients according to the number of FISH alterations. Patients with no abnormalities (41.2%) had the most favorable outcomes (median OS 78.3 months), while those with ≥2 abnormalities showed progressively worse survival, supporting the concept that cytogenetic complexity is a key prognostic factor [19].

In our cohort of high-risk patients defined according to the IMWG/IMS HR criteria, the use of modern first-line therapies, including anti-CD38 monoclonal antibodies, immunomodulatory drugs, and proteasome inhibitors, resulted in high response rates, emphasizing the importance of optimized induction regimens.

When comparing our results with international data, several key observations emerge. The prevalence and survival outcomes of del(17p) in our cohort are consistent with published data, supporting the external validity of our findings. However, the presence of co-occurring cytogenetic abnormalities, particularly involving chromosome 1, significantly worsened outcomes, confirming the concept of double-hit and triple-hit myeloma [15]. While overall response rates remained high across most subgroups (generally >80%), relapse rates and survival outcomes were strongly influenced by cytogenetic risk burden. These findings highlight that depth of response alone is insufficient to overcome adverse cytogenetic biology, emphasizing the need for risk-adapted therapeutic strategies and closer monitoring in high-risk patients.

In our cohort of HR patients, according to the IMWG/IMS criteria, the use of modern therapies based on anti-CD38 antibodies, IMiDs, and PIs in first-line treatment resulted in high response rates, underscoring the importance of administering these agents in optimal combinations. An important aspect of our analysis is the evaluation of the impact of single ASCT versus TANDEM ASCT on survival in HR patients, according to the new IMWG/IMS criteria. In the analyzed cohort, of the 103 patients classified as HR according to IMWG/IMS, 35 patients underwent a single ASCT procedure, and 10 received a TANDEM transplant. Further analysis did not reveal any benefits regarding OS or PFS in patients who received two ASCT procedures in TANDEM. Similar results were also obtained in the cytogenetic HR cohort defined based on the old criteria, prior to the reclassification of patients as HR according to IMWG/IMS (where median OS was not reached in patients with a single ASCT procedure vs. a median OS of 52.8 months in patients with TANDEM). These results are consistent with data in the literature, which underscore the idea that in the era of induction therapies based on quadruple therapy and maintenance, the indication for performing a second autologous transplant in TANDEM should be applied to a selected group of patients, given that it does not provide major benefits [21,22]. Furthermore, a meta-analysis conducted in 2020 on the results of the CASSIOPEIA, ALCYONE, and MAIA trials demonstrated that adding daratumumab to the treatment regimen has the potential to improve prognosis, even in patients considered high-risk (HR) [23]. Although modern quadruplet regimens incorporating anti-CD38 monoclonal antibodies have significantly improved response depth in HR-MM, recent studies such as OPTIMUM and CONCEPT continue to support the importance of intensified and prolonged treatment strategies, including consolidation and maintenance approaches, particularly in biologically ultra-high-risk disease [24,25]. Another important aspect in current practice is the depth of the responses obtained, particularly after first-line treatment, given their prognostic significance. In our patient cohort, a trend toward deeper responses was observed in the group of patients who underwent only one ASCT procedure. These results are consistent with data from a real-world Canadian study, where performing ASCT in TANDEM did not improve outcomes for patients with HRMM [26].

Therefore, the results obtained in our cohort, which are consistent with those in the literature, support the conclusion that adding a second autologous transplant procedure in the TANDEM regimen does not provide additional benefits for all HR patients. However, given the small cohort of patients who underwent the TANDEM regimen, the results should be interpreted with caution.

An important strength of our study is the application of the recently updated IMWG/IMS high-risk definition in a large real-world Eastern European cohort. Unlike highly selected clinical trial populations, our cohort reflects routine clinical practice, including heterogeneous treatment strategies, variable access to comprehensive cytogenetic testing, and real-life implementation challenges associated with modern risk stratification. Our findings, therefore, provide clinically relevant insight into the feasibility and prognostic impact of the updated classification system outside controlled trial settings. In addition, the present study highlights the importance of cytogenetic complexity and chromosome 1 abnormalities within the contemporary definition of high-risk disease. Despite high response rates achieved with modern therapeutic combinations, adverse biological profiles continued to be associated with inferior progression-free and overall survival, emphasizing that current therapies only partially overcome high-risk disease biology.

## 4. Limitations

Our study has several limitations that should be acknowledged. First, due to the retrospective real-world design, complete characterization of all high-risk cytogenetic abnormalities (HRCAs) included in the updated IMWG/IMS definition was not uniformly available for all patients. Although FISH analysis was available in 408 patients, TP53 mutational testing was not systematically performed, monoallelic versus biallelic del(1p) could not be consistently differentiated, and chromosome 1 abnormalities were not uniformly subclassified as gain versus amplification. In addition, although del(17p) percentages were available in patients classified as high risk, low-level subclonal TP53 deletions may have been underrepresented due to the applied classification thresholds. Missing cytogenetic data were not imputed, and all analyses were performed using available-case data only. Second, only 408 of the 738 initially diagnosed patients had baseline FISH evaluation available. This reflects real-world limitations related to sample quality, plasma cell enrichment feasibility, logistical availability of cytogenetic testing, and the progressive implementation of standardized molecular diagnostics during the study period. Potential differences between patients with and without available FISH data may therefore have introduced a degree of selection bias.

Third, several cytogenetic subgroups were represented by a limited number of patients, particularly those involving rare combinations of chromosome 1 abnormalities and IGH translocations. Consequently, subgroup-specific prognostic observations should be interpreted cautiously, and no definitive conclusions can be drawn regarding the biological behavior of these rare entities. In particular, the apparently favorable outcomes observed in the small del(1p) subgroup should not be interpreted as evidence of a less aggressive disease course, especially given previously published larger datasets demonstrating the adverse prognostic impact of del(1p).

Despite these limitations, an important strength of our study is the application of the recently updated IMWG/IMS high-risk definition in a large real-world Eastern European cohort. Unlike clinical trial populations, our cohort reflects routine clinical practice, including heterogeneous treatment strategies, variable access to comprehensive cytogenetic testing, and real-life implementation challenges. These findings therefore provide clinically relevant insight into the feasibility and prognostic impact of modern high-risk stratification outside controlled trial settings.

## 5. Materials and Methods

### 5.1. Patients and Study Design

We conducted a single-center, retrospective study evaluating 738 patients diagnosed with multiple myeloma over an 8-year period (2017–2025) to characterize their cytogenetic profile using Fluorescence In Situ Hybridization (FISH) at the time of diagnosis. The included patients were analyzed in terms of high-risk cytogenetic abnormalities and for the impact of these alterations on survival outcomes and treatment response.

Patient selection was based on the following inclusion criteria: confirmed diagnosis of multiple myeloma and availability of FISH analysis performed on bone marrow plasma cells (CD138+ selection) at the time of diagnosis. We excluded from the study all patients for whom FISH analysis was unavailable or who had del(17p) < 20%.

The diagnostic criteria for multiple myeloma and the response and relapse criteria used in the analysis were those recommended by the International Myeloma Working Group (IMWG) (14), valid at the time of each patient’s evaluation.

### 5.2. Data Collection

Patient data were extracted from the Romanian National Multiple Myeloma Registry database, with only patients diagnosed and monitored at the Hematology Clinic of the Fundeni Clinical Institute selected. Information was collected on FISH test results, treatment types and responses, and data on disease progression and overall survival. The analysis of these data aimed to evaluate the impact of specific cytogenetic abnormalities and their combinations on disease progression and survival.

### 5.3. Ethics Statement

The study was conducted in accordance with the Declaration of Helsinki. All information was collected retrospectively with the approval of the Ethics Committee of the Fundeni Clinical Institute (No. 12790/03/13/2026). The patient’s informed consent for the use of medical data for research purposes was documented in the patient’s medical records, in accordance with institutional procedures.

### 5.4. FISH Analysis

Plasma cells (CD138+) were isolated from the bone marrow aspirates of all patients included in the study. Initially, the number of CD138+ cells in the bone marrow aspirates was estimated by flow cytometry. Subsequently, plasma cells were extracted from heparinized bone marrow aspirates (5 mL) using the MACS protocol (Miltenyi Biotec, Bergisch Gladbach, Germany), according to the manufacturer’s instructions. Bone marrow mononuclear cells were previously separated using a density gradient with Ficoll–Paque. The CD138+ plasma cells were magnetically labeled with CD138 MicroBeads and loaded onto MACS columns placed within the magnetic field of a MACS Separator. Magnetic labeling was performed on at least 1 × 10^6^ cells per sample. For samples intended for FISH analysis, the enriched plasma cells were treated with KCl and a fixative solution (methanol: acetic acid, 3:1). The most frequent chromosomal abnormalities observed in multiple myeloma were evaluated by interphase FISH. Specific probes were used to detect the most relevant alterations: TP53/D17Z1 for del(17p), CKS1B(1q21.3)/CDKN2C(1p32.3) for chromosome 1 abnormalities, dual-color probes for translocation t(14;16) (MAF(16q23)/IGH(14q32.3)) and t(4;14) (FGFR3(4p16)/IGH(14q32.3)) (CytoCell^®^, Oxford Gene Technology, Cambridge, UK). Samples were denatured at 75 °C using Hychrome (Euroclone, Pero, Italy) and hybridized at 37 °C for 16 h. To determine cut-off values, 100 nuclei were examined, and the upper limits of normal were set at 10% for the entire panel. Images were acquired using an Olympus BX41 fluorescence microscope (Olympus Corporation, Tokyo, Japan), and results were reported according to ISCN 2024 standards.

For del(17p), the laboratory positivity threshold for FISH detection was established at 10%; however, patients were classified as high risk only when ≥20% of analyzed nuclei carried the abnormality, according to the study classification criteria.

### 5.5. Statistical Analysis

Data processing, statistical analysis, and graphical visualization were performed using Microsoft Power BI Desktop (version 2.151.1182.0, 64-bit; Microsoft, Redmond, WA, USA). Overall survival (OS) and progression-free survival (PFS) were estimated using the Kaplan–Meier method. OS was defined as the time from diagnosis to death from any cause, while PFS was defined as the time from the initiation of first-line therapy to documented disease progression or death from any cause. This definition was applied consistently across the entire cohort, including patients who underwent single or tandem ASCT, as transplantation was considered part of the first-line treatment strategy. Median follow-up was calculated using the reverse Kaplan–Meier method, where censored dates are treated like events and actual OS events are censored.

High-risk cytogenetic abnormalities were defined based on FISH analysis results and in accordance with the updated high-risk criteria from the IMWG/IMS guidelines (8). In our study, we classified as HR patients those who, upon FISH analysis, showed one of the following: the presence of del(17p) at a minimum of 20%; or the presence of one of t(4;14), t(14;16), or t(14;20) associated with changes in chromosome 1. We also included in the high-risk category patients with a B2M value ≥ 5.5 mg/L in the presence of a normal creatinine value (<1.2 mg/dL). The application of risk criteria according to IMWG/IMS recommendations in the study cohort was limited by the lack of differentiation between the monoallelic and biallelic status of del(1p) and by the absence of TP53 testing in all patients; although TP53 analysis was recently initiated, the data obtained are not sufficiently mature to be included in the final analysis. Complete characterization of all high-risk cytogenetic abnormalities (HRCAs) included in the updated IMWG/IMS definition was not uniformly available for all patients. In particular, TP53 mutational status was not systematically assessed, and monoallelic versus biallelic del(1p) could not be consistently differentiated. In addition, chromosome 1 abnormalities were not uniformly subclassified as gain versus amplification in all cases. Missing cytogenetic data were not imputed, and all analyses were performed using available-case data only.

Patients classified as HR according to IMWG/IMS were subsequently stratified based on the number of high-risk cytogenetic abnormalities. Patients with a single cytogenetic abnormality were considered single-hit, those with two concurrent abnormalities were classified as double-hit, and those with three or more high-risk cytogenetic abnormalities were defined as triple-hit. The analysis of these subgroups examined the impact these associations had on survival and treatment response.

## 6. Conclusions

In conclusion, our real-world analysis confirms the significant prognostic impact of high-risk cytogenetic abnormalities in multiple myeloma. Del(17p) remains the most frequent high-risk lesion, while combinations involving chromosome 1 abnormalities and high-risk translocations define double-hit and triple-hit subgroups with particularly poor outcomes.

Although modern therapies and ASCT achieve high response rates, both progression-free and overall survival remain strongly dependent on cytogenetic risk complexity.

Importantly, in our cohort, TANDEM ASCT did not provide additional survival benefit, supporting a more selective approach to treatment intensification in the era of novel agents.

These findings underscore the need for risk-adapted treatment strategies, careful patient selection, and the development of innovative therapeutic approaches to improve long-term outcomes in high-risk multiple myeloma.

## Figures and Tables

**Table 1:** Distribution of high-risk patients according to IMS/IMWG HR criteria (n = 408).

High-Risk Feature	Patients, n (%)
del(17p)	60 (15%)
t(4;14) and 1q+	10(2.5%)
t(14;16) and 1q+	2 (1%)
del(1p) and 1q+	2 (1%)
del(1p)	5 (1%)
B2M	33 (8%)

**Table 2:** Baseline characteristics of the HR population (n = 103).

Characteristic		n (%)
Age (years)		
	Median (range)	60 (38–84)
	≤65 years, n (%)	74 (72%)
Sex		
	Male	53 (51%)
	Female	50 (49%)
ISS stage		
	I	17 (17%)
	II	18 (17%)
	III	68 (66%)
Myeloma subtype		
	Ig G	61 (59%)
	Ig A	20 (20%)
	Ig D	1 (1%)
	Light chain only	21 (20%)
Light chain type		
	κ	12 (57%)
	λ	9 (43%)

**Table 3:** Treatment response according to transplantation strategy: single versus TANDEM ASCT.

Response	1 × ASCT (n = 35)	TANDEM ASCT (n = 10)	*p* Value *
CR	13 (37%)	2 (20%)	0.4556
VGPR	14 (40%)	6 (60%)	0.301
PR	7 (20%)	1 (10%)	0.6611
ORR (≥PR)	34 (97%)	9 (90%)	0.399
Relapse rate	26%	40%	0.2056
Overall survival rate at 12 months	86%	90%	0.2516

* *p*-value is calculated using the Fisher Exact test for response groups proportions (response group vs. other responses) or Log-rank tests for PFS (relapse rate) and OS (Overall survival rate at 12 months).

**Table 4:** Comparison of HR cytogenetic subgroups.

Combination	n	Prevalence (%)	Median OS (Months)	Median PFS (Months)	ORR (%)	Relapse Rate (%)	Survival Rate (12 Months)
All cases	408	100.00%	71.1	25	88.1	52.7	87.40%
Standard risk	305	74.80%	68.4	28	90.3	52.8	88.70%
*del17p*	60	14.70%	52.9	18	85.2	46.7	82.00%
isolated *del17*	32	7.80%	85.8	18	87.1	50	82.20%
*del(1p)*	5	1.20%	NR	NR	100	20	80.00%
*1q+* and *del(1p)*	2	0.50%	1	0	100	50	50.00%
*t(4;14)* with *1q+/del(1p)*	10	2.50%	NR	13	66.7	40	74.10%
*t(14;16)* with *1q+/del(1p)*	2	0.50%	NA	NA	0	100	NA
B2M ≥ 5.5, Creatinine <1.2-all	41	10.00%	85.8	22	78.4	65.9	84.70%

Table legend: B2M expressed in mg/L; creatinine expressed in mg/dL.

## Data Availability

All data concerning the publication of this article can be requested from the corresponding author.

## Abbreviations

The following abbreviations are used in this manuscript:

amp(1q)	amplification of chromosome 1q (≥4 copies)
ASCT	autologous stem cell transplantation
B2M	beta-2 microglobulin
CR	complete response
CyBorD	cyclophosphamide, bortezomib, dexamethasone
Dara-VRd	daratumumab, bortezomib, lenalidomide, dexamethasone
del(1p)	deletion of the short arm of chromosome 1
del(17p)	deletion of the short arm of chromosome 17
DRd	daratumumab, lenalidomide, dexamethasone
EHA	European Hematology Association
EMN	European Myeloma Network
FISH	fluorescence in situ hybridization
gain(1q)	gain of chromosome 1q (3 copies)
HR	high risk
IGH	immunoglobulin heavy chain
IMWG	International Myeloma Working Group
LDH	lactate dehydrogenase
MM	multiple myeloma
MRD	minimal residual disease
NE	not estimable
NR	not reached
ORR	overall response rate
OS	overall survival
PAD	bortezomib, doxorubicin (adriamycin), dexamethasone
PFS	progression-free survival
PR	partial response
RAD	lenalidomide, doxorubicin (adriamycin), dexamethasone
R-ISS	Revised International Staging System
SR	standard risk
t(4;14)	translocation between chromosomes 4 and 14
t(14;16)	translocation between chromosomes 14 and 16
TANDEM ASCT	tandem autologous stem cell transplantation (two consecutive transplants)
TP53	tumor protein p53
VGPR	very good partial response
VelDex	bortezomib (velcade), dexamethasone
VMP	bortezomib, melphalan, prednisone
VTD	bortezomib, thalidomide, dexamethasone
VRd	bortezomib, lenalidomide, dexamethasone
VRd-lite	dose-attenuated bortezomib, lenalidomide, dexamethasone regimen
1q+	gain or amplification of chromosome 1q
